# Identification of determinant factors for crash severity levels occurred in Addis Ababa City, Ethiopia, from 2017 to 2020: using ordinal logistic regression model approach

**DOI:** 10.1186/s12889-023-16785-3

**Published:** 2023-09-29

**Authors:** Tariku Bekelcho, Ararso Baru Olani, Asfawosen Woldemeskel, Micheal Alemayehu, Geleta Guta

**Affiliations:** 1 Department of Emergency Medicine and Critical Care, Arba Minch University, Arba Minch, Ethiopia; 2 Department of Medicine, Ethiopian Police University, Sendafa, Ethiopia; 3 Department of Emergency and Critical Care, Tirunesh Beijing General Hospital, Addis Ababa, Ethiopia; 4 Department of Water Resources and Irrigation Engineering, Arba Minch University, Arba Minch, Ethiopia

**Keywords:** Road traffic injuries, Ordinal logistic regression, Injury severity level, Addis Ababa, Ethiopia

## Abstract

**Background:**

Road traffic Injuries (RTI) are multifaceted occurrences determined by the combination of multiple factors. Also, severity levels of injuries from road traffic accidents are determined by the interaction of the composite factors. Even though most accidents are severe to fatal in developing countries, there is still a lack of extensive researches on crash severity levels and factors associated with these accidents. Hence, this study was intended to identify severity levels of road traffic injuries and determinant factors in Addis Ababa City, Ethiopia.

**Methods:**

The study was conducted in Addis Ababa, the capital city of Ethiopia, using secondary data obtained from the Addis Ababa Police Commission office. The ordinal logistic regression model was used to investigate road traffic injury severity levels and factors worsening injury severity levels using the recorded dataset from October 2017 to July 2020.

**Results:**

A total of 8458 car accidents were considered in the study, of which 15.1% were fatal, 46.7% severe, and 38.3% were slight injuries. The results of the ordinal logistic regression analysis estimation showed that being a commercial truck, college and above level educated driver, rollover crash, motorbike passengers, the crash day on Friday, and darkness were significantly associated factors with crash injury severity levels in the study area. On the contrary, driving experience (> 10 years), passenger of the vehicle, two-lane roads, and afternoon crashes were found to decrease the severity of road traffic injuries.

**Conclusions:**

Road traffic injury reduction measures should include strict law enforcement in order to maintain road traffic rules especially among commercial truckers, motorcyclists, and government vehicle drivers. Also, it is better to train drivers to be more alert and conscious in their travels, especially on turning and handling their vehicles while driving.

## Introduction

Road traffic injuries are a global disaster and among the top public health problems in recent years. Mortality from the road traffic accidents on the world remains high, with an estimated 1.35 million people dying each year worldwide which ranking it as the eighth leading cause of death for all age groups and the leading cause of death for children and young adults aged 5–29 years [1]. Among world regions, low-income countries share a larger amount of road traffic fatality rate (29.2/100,000 population), out of which Africa accounts for 20% of global road traffic deaths with nearly 272 000 deaths. Also, African RTIs have a significant number of the accidents under reported, mainly nonfatal injuries [1, 2]. From a total of the world’s RTI fatalities, 90% occurs in low- and middle-income countries. Even though these low- and middle-income countries have a low number of registered motor vehicles, the number of road traffic deaths is alarmingly higher than in other countries. For instance, in low-income African countries, the number of registered motor vehicles in 2016 was only 27%, however, the number of road traffic deaths is substantially higher, accounting for 59% [3, 4].

Statistics also show the disparities in road traffic deaths by the types of road users. Among the worlds’ road users, pedestrians and cyclists account for 26% of all road traffic deaths, unfortunately, Africa and Eastern Mediterranean pedestrians and cyclists’ mortality rates are as high as 44% and 36% respectively [5]. RTIs causes not only victims’ injuries and deaths, but also very significant amount of economic loss which is estimated to cost around United States Dollar (USD) 1,800 billion, or 3% of Gross Domestic Products (GDP) globally, while in low- and middle-income countries, economic losses are about 5% of their GDP per year. Moreover, road traffic crashes are predicted to become the seventh leading cause of death by 2030 unless continuous and sustained efforts and actions are taken to reduce road traffic accidents [4].

Despite an increase in the overall number of deaths and burden of the RTIs, the road safety issue was undermined and neglected, which does not receive the due attention it deserves. However, after extensive interventional measures taken in middle-and high-income countries, these measures have effectively reduced road traffic deaths in these countries, even though not a single low-income country has reduced in overall deaths and burden of RTIs [5, 6].

Having a better understanding of the contributing factors for road traffic injuries is very fundamental for road safety policies and crash prediction. Nevertheless, road traffic injuries are complex community health problems that are caused by the combination and interaction of multiple factors that make full understanding of the event and better insight toward the problem is challenging. So far, illegal drivers’ behavior, vehicle features, roadway characteristics, and other environmental conditions have been reported as major determinant factors [7].

In the literature, road traffic accident injury severity levels are determined by a composite factors and conditions. As mentioned, severe injuries or deaths were higher in traffic crashes with pedestrians at fault, drivers’ illegal behaviors, vehicles with unsafe status and crashes occurring at night with street lighting were some of the reported factors [8]. From the vast majority of pedestrian faults, crossing the road by violating the road traffic laws such as crossing outside of designated crossing areas was commonly reported [9].

In Ethiopia, there is a rapid growth in motorization and road infrastructure that has brought alarming road safety concerns in recent years. As reported by scholars, on average, around 13.34% of motorized vehicles and 10.4% of asphalt road development were registered as yearly growth rates. In the same study, on average, yearly around 9.28%, 11.39%, 10.76% and 5% were registered as fatal, serious and slight injuries, and property damage, respectively. Also, the economic losses due to RTIs in Ethiopia are estimated to be around 3.3 billion Birr per year due to the occurrences of road traffic accidents [10].

In the literature, scholars have reported focusing on identifying contributing factors of road traffic accidents and ways forwards [11].

Studies conducted in Ethiopia indicate that factors such as a younger driver, a less educated driver, being male, younger road users, driver’s experience, drunk and speedy driving, a motorcyclist or passenger without a helmet, vehicle size, dark lighting conditions, and a vehicle occupant traveling unrestrained in the back of a truck are major factors contributing to the RTI occurrences and worsening the injury severity levels [11–14].

Few road safety measures such as; increasing the number of speed breakers, strict enforcement of traffic rules, the imposition of heavy fines and punishments on drivers who violated the traffic rules, and striving to increase the growth rate of road infrastructures, etc. have been initiated in Ethiopia recently. However, the road traffic accidents induced mortality and occurrences of fatal injuries were increasing despite all these activities done so far [14, 15].

However, it has been reported that there is an urgent need to design a predictive model for road traffic accidents or crash severity. This will help manage these tragic accidents and improve road safety because predictive models can determine the frequency of accidents and the severity level of crashes [16]. Also, for an outcome that may be thought of as ordinal, it is often preferable to use all of the ordinal values, rather than condensing them into fewer categories or dichotomizing variables. It has been shown that collapsing categories reduces statistical power [17].

As a result, the ordinal regression model is an appropriate model for outcomes that can be considered ordinal because it is more sensitive to the ordinality of the data and has stable parameter estimates [18]. Thus, the outcome variable for the current study is road traffic injury severity levels, and categorized as slight, severe, and fatal injuries is ordinal data in nature.

Consequently, the current study was intended to assess injury severity levels induced by road traffic accidents and associated factors in Addis Ababa, Ethiopia.

## Methods

### Data description

The crash data used in this study were obtained from the Addis Ababa city police commission road safety department. The reported data from October 2017 to July 2020 were used. The collected and recorded road traffic accident data comprised accident circumstances, the severity of injuries, immediate outcomes of an accident, and property damage. Data were collected on an excel sheet using variables such as; date and time of an accident, biographic data of the victims such as: age, gender, drivers’ education, relationship with the car, drivers’ experience, location of injury, type of roads, injury severity outcome, pedestrian victim, type of road, type of vehicle, and environmental conditions (weather and air condition, light condition). The Collected data were exported into R software for the statistical analysis.

The original data set categorized injury severity levels into slight, severe, and fatal [19, 20]. Traffic police crash accident reports at accident scenes do not narrate or describe injuries in detail. This is because of the lack of traffic police qualifications and training on trauma assessment and management. In addition,, trauma experts needed to designate injury severity levels are not available in the crash scenes. Likewise, medical reports are hard to obtain because it is difficult to find out linked traffic police and medical patient charts at the same place. The result was that this study relied on the original classifications of slight, severe, and fatal injuries recorded on the traffic police reports [21]. This is similar to some previous studies that classified injuries in the same way [16, 22].

For the current study, the outcome variable was crash injury severity levels (Fatal, Severe, and Slight). For such ordered outcome variables, it is appropriate to analyze with an ordinal logistic regression model, which is a special type of the generalized linear model, and related to a response variable that follows the exponential family via an appropriate link function [23]. Whereas, the independent variables for road crash injury severity levels include: type of vehicle (two or three wheelers, public transport, commercial truck, automobile, and others), victims of crash (Pedestrian, Passenger, Driver of vehicle, Motorcyclist, Motor passenger), type of road (Straight, Zigzag, Curve, Tilted, Hill), and owner of the vehicle (Private, Government), etc.

### Statistical analysis

The chi-square test (Χ^2^) of independence was used to determine the inclusion of variables in the model for further analysis (Table 3). The ordinal logistic regression model was used to assess an association between outcome and explanatory variables. The variables that have a statistically significant association with road crash severity level at 5% significance level were used for further analysis using the ordinal logistic regression model (Table 4).

### Model description

The ordinal logistic regression model is a special type of the generalized linear model, which is appropriate for analysis of the ordered responses without losing information from collapsing (or ignoring) some categories of the ordered responses by typically maintaining statistical power. While analyzing data with this model, it would take into account the ordinal nature of the outcome, and an estimated odds ratio may address the questions asked of the analysis [24].

In other words, it takes into account the ordering of the categories of the response variable(s). In this model, we consider the probability of an individual event and all others above it in the ordinal ranking. Cumulative probabilities rather than probabilities for discrete categories are concerned. The goal of such a cumulative odd model is to simultaneously consider the effects of a set of explanatory variables across these possible consecutive cumulative splits in the outcome. One probability is monotonically increasing or decreasing as a function of x. Curves of probabilities for intermediate categories are unimodal with the modes (maximum) corresponding to the order of the categories. The response category does not affect conclusions regarding the relationship between y and x. Therefore, the specific combination of categories examined does not lead to substantially different conclusions regarding the relationship between responses and x [25].

This study applied the ordinal logistic regression model, relating injury severity to potential risk factors. Let Y represents an ordinal response variable with k ordered outcomes, and X represents a vector of explanatory variables, then the ordered logit model describing the relationship between these variables can be described [20, 22, 26]:

1 $$\mathrm{logit}\lbrack \,P\left(\mathrm y\leq\mathrm j\right)=\log\left\lfloor\frac{P\left(y\leq j\right)}{1-P\left(y\leq j\right)}\right\rfloor=\mathrm\alpha+\mathrm\beta\mathrm X,\,\mathrm{for\,j}=1,2,\,\dots,\mathrm k-1$$

Where:

*P*(y ≤ j) = *P1* + *P2* + … + *Pj* = are the cumulative probabilities,

αj = intercept parameter (threshold parameter) of the cumulative probability j,

β = is a column vector of parameters that describe the effects of the explanatory variable(s) on the dependent variable.

From model (1), since the relationship between all pairs of categories is the same, we obtain only one coefficient (beta) for the all the categories in the estimated model and different intercepts (alpha) for each category. Model (1) can then be expressed in terms of cumulative probabilities as [16, 26, 27]:

2 $$\mathrm{P}\left(\mathrm{y}\le \mathrm{j}\right)=\frac{\mathrm{exp}\left(\alpha j+\beta X\right)}{1+\mathrm{exp}\left(\alpha j+\beta X\right)},\mathrm{\, for\, j}=1, 2,\dots ,\mathrm{k}-1$$

The parameters in the model (2) are estimated using the maximum likelihood estimation method [26, 28].

Since we cannot observe y*, instead we can only observe the N levels of the response through the following structural model:

3 $$\mathrm y=\left\{\begin{array}{l}1\;if\;y\ast<\tau1\\2\;if\;\tau1<y\ast\leq\tau2\\3\;if\;\tau2<y\ast\leq\tau3\\.\\.\\N\;if\;\tau N<y\ast\end{array}\right.$$

Where the parameters; τi are the externally imposed endpoints of the observable categories. Then the ordered logit technique will use the observations on y, which are a form of censored data on y*, to fit the parameter vector β [22, 26, 29]. Also, each level of y has its own intercept, which is the negative of the corresponding threshold, but there is a common coefficient vector $\beta$ [16, 22, 29].

### The proportional odds assumption

This assumption states that, parameters should not change for different categories, or the correlation between the independent variable and dependent variable does not change for dependent variable categories. In other words, the effects of any explanatory variables are consistent across the different categories of the outcome variable. In ordinal logistic regression, there will be separate intercept terms at each threshold, but a single odds ratio (OR) for the effect of each explanatory variable [22, 24, 28, 29]. The proportional odds assumption for this study was tested and presented in (Tables 1 and 2).

**Table 1:** Test of parallel lines

Model	-Log likelihood	Chi-square	df	Sig.
Null hypothesis	8528.88			
General	7457.98	15,024.84	8400	0.217

**Table 2:** Goodness of fit

	Chi-square	df	Sig.
Pearson	16,314.5	8456	0.897
Deviance	16,272.9	8456	0.997

We have used R software version 4.2.3 for the analysis using the polr () function in the MASS package. The vglm () function in the VGAM package was used to fit various of threshold-based models to the ordinal data. The po test function in R implements tests in the MASS package and was used to fit the model. Also, ANOVA function to perform an ANOVA test was used to determine if the slope coefficients are equal for all levels of the response variable [16, 30–32].

## Results

### Sociodemographic characteristics of the study subjects

Out of the total number of 8458 accidents sustained, most drivers (86.4%) were male and in the age group of the 18–30 years which accounts for 46%. From the victims of the crash, pedestrians were dominant, accounting for 71.4%, followed by passengers of the vehicle 18.2% (Table 3).

**Table 3:** Distribution of road traffic accident injury severity levels in Addis Ababa, Ethiopia from October 2017–July 2020

**Variables**	**Categories**	**Accident injury severity levels frequencies**				
		**Fatal injury** 1274 (15.1%)	**Severe injury** 3947 (46.7%)	**Slight injury** 3237 (38.3%)	Total 8458 (100%)	*p*-value
Driver sex	Male	1166 (13.8%)	3282 (38.8%)	2859 (33.8%)	7307 (86.4%)	.000 ***
	Female	108 (1.3%)	665 (7.9%)	378 (4.5%)	1151 (13.6%)	
Driver’s age	< 18 years	20 (0.2%)	39 (0.5%)	67 (0.8%)	126 (1.5%)	.016*
	18–30 years	577 (6.8%)	1837 (21.7%)	1479 (17.5%)	3893 (46.0%)	
	31–50 years	592 (7.0%)	1794 (21.2%)	1482 (17.5%)	3868 (45.7%)	
	> 50 years	85 (1.0%)	277 (3.3%)	209 (2.5%)	571 (6.8%)	
Driver educational level	High school and lower	466 (5.5%)	2684 (31.7%)	2118 (25.0%)	5268 (62.3%)	.000***
	College and above	808 (9.6%)	1263 (14.9%)	1119 (13.2%)	3190 (37.7%)	
Day of crash	Monday	198 (2.3%)	591 (7.0%)	531 (6.3%)	1320 (15.6%)	.000***
	Tuesday	182 (2.2%)	529 (6.3%)	465 (5.5%)	1176 (13.9%)	
	Wednesday	173 (2.0%)	560 (6.6%)	501 (5.9%)	1234 (14.6%)	
	Thursday	154 (1.8%)	646 (7.6%)	497 (5.9%)	1297 (15.3%)	
	Friday	230 (2.7%)	359 (4.2%)	303 (3.6%)	892 (10.5%)	
	Saturday	137 (1.6%)	655 (7.7%)	491 (5.8%)	1283 (15.2%)	
	Sunday	199 (2.4%)	607 (7.2%)	449 (5.3%)	1255 (14.8%)	
Driving experience in years	1–2	748 (8.8%)	2079 (24.6%)	1704 (20.2%)	4531 (53.6)	.000***
	3–5	372 (4.4%)	638 (7.5%)	539 (6.4%)	1549 (18.3)	
	6–10	98 (1.2%)	487 (5.8%)	396 (4.7%)	981 (11.6%)	
	> 10	56 (0.7%)	743 (8.8%)	598 (7.1%)	1397 (16.5%)	
Type of crash	Crash with pedestrian	1001 (11.8%)	2930 (34.6%)	2147 (25.4%)	6078 (71.9%)	.000***
	Crash with another vehicle	138 (1.6%)	666 (7.9%)	766 (9.1%)	1570 (18.6%)	
	Roll over	62 (0.7%)	130 (1.5%)	122 (1.4%)	314 (3.7%)	
	Crash with animal or objects	51 (0.6%)	143 (1.7%)	141 (1.7%)	335 (4.0%)	
	Others	22 (0.3%)	78 (0.9%)	61 (0.7%)	161 (1.9%)	
Type of vehicle	Two or three wheelers	76 (0.9%)	591 (7.0%)	539 (6.4%)	1206 (14.3%)	.000***
	Public transport	333 (3.9%)	1286 (15.2%)	989 (11.7%)	2608 (30.8%)	
	Commercial truck	487 (5.8%)	1119 (13.2%)	897 (10.6%)	2503 (29.6%)	
	automobile	233 (2.8%)	861 (10.2%)	761 (9.0%)	1855 (21.9%)	
	Others	145 (1.7%)	90 (1.1%)	51 (0.6%)	286 (3.4%)	
The owner of the vehicle	Private	990 (11.7%)	3223 (38.1%)	2766 (32.7%)	6979 (82.5%)	.000***
	Government	200 (2.4%)	507 (6.0%)	304 (3.6%)	1011 (12.0%)	
	Others	84 (1.0%)	217 (2.6%)	167 (2.0%)	468 (5.5%)	
Movement of vehicle	Straight	989 (11.7%)	3182 (37.6%)	2683 (31.7%)	6854 (81.0%)	.000***
	Curved	158 (1.9%)	499 (5.9%)	409 (4.8%)	1066 (12.6%)	
	Reverse	30 (0.4%)	125 (1.5%)	110 (1.3%)	265 (3.1%)	
	Parked	42 (0.5%)	58 (0.7%)	35 (0.4%)	135 (1.6%)	
	Other	55 (0.7%)	83 (1.0%)	0 (0.0%)	138 (1.6%)	
Victims of crash	Pedestrian	1017 (12.0%)	2862 (33.8%)	2160 (25.5%)	6039 (71.4%)	.000***
	The passenger of vehicle	151 (1.8%)	706 (8.3%)	686 (8.1%)	1543 (18.2%)	
	The driver of vehicle	65 (0.8%)	200 (2.4%)	262 (3.1%)	527 (6.2%)	
	Motorcyclist	21 (0.2%)	134 (1.6%)	105 (1.2%)	260 (3.1%)	
	Motor passenger	20 (0.2%)	45 (0.5%)	24 (0.3%)	89 (1.1%)	
Age of the victims	< 18 years	102 (1.2%)	992 (11.7%)	802 (9.5%)	1896 (22.4%)	.000***
	18–30 years	398 (4.7%)	1192 (14.1%)	1090 (12.9%)	2680 (31.7%)	
	31–50 years	503 (5.9%)	939 (11.1%)	787 (9.3%)	2229 (26.4%)	
	> 51 years	271 (3.2%)	824 (9.7%)	558 (6.6%)	1653 (19.5%)	
Sex of victims	Male	969 (11.5%)	2743 (32.4%)	2235 (26.4%)	5947 (70.3%)	.000***
	Female	305 (3.6%)	1204 (14.2%)	1002 (11.8%)	2511 (29.7%)	
Movement of Pedestrians	Crossing	694 (8.2%)	2179 (25.8%)	1596 (18.9%)	4469 (52.8%)	.000***
	Walking/standing	308 (3.6%)	1483 (17.5%)	1411 (16.7%)	3202 (37.9%)	
	Sitting	72 (0.9%)	260 (3.1%)	226 (2.7%)	558 (6.6%)	
	Other	200 (2.4%)	25 (0.3%)	4 (0.0%)	229 (2.7%)	
Sub-city of the accidents	Akaki Kality	158 (1.9%)	332 (3.9%)	329 (3.9%)	819 (9.7%)	.000***
	Arada	66 (0.8%)	431 (5.1%)	40 (0.5%)	537 (6.3%)	
	Nifas Silk	187 (2.2%)	735 (8.7%)	831 (9.8%)	1753 (20.7%)	
	Bole	226 (2.7%)	736 (8.7%)	502 (5.9%)	1464 (17.3%)	
	Yeka	180 (2.1%)	203 (2.4%)	148 (1.7%)	531 (6.3%)	
	Kolfe Keraniyo	156 (1.8%)	515 (6.1%)	398 (4.7%)	1069 (12.6%)	
	Addis Ketema	76 (0.9%)	238 (2.8%)	252 (3.0%)	566 (6.7%)	
	Kirkos	75 (0.9%)	373 (4.4%)	273 (3.2%)	721 (8.5%)	
	Gulele	63 (0.7%)	90 (1.1%)	292 (3.5%)	445 (5.3%)	
	Lideta	87 (1.0%)	293 (3.5%)	172 (2.0%)	552 (6.5%)	
Division of roads	Round about	766 (9.1%)	1615 (19.1%)	1174 (13.9%)	3555 (42.0%)	.000***
	One lane	89 (1.1%)	341 (4.0%)	324 (3.8%)	754 (8.9%)	
	Two lanes	311 (3.7%)	1696 (20.1%)	1435 (17.0%)	3442 (40.7%)	
	Others	108 (1.3%)	295 (3.5%)	304 (3.6%)	707 (8.4%)	
The type of road	Straight	1020 (12.1%)	2695 (31.9%)	2202 (26.0%)	5917 (70.0%)	.000***
	Zigzag	29 (0.3%)	24 (0.3%)	25 (0.3%)	78 (0.9%)	
	Curve	87 (1.0%)	1005 (11.9%)	833 (9.8%)	1925 (22.8%)	
	Tilted	60 (0.7%)	137 (1.6%)	86 (1.0%)	283 (3.3%)	
	Hill	78 (0.9%)	86 (1.0%)	91 (1.1%)	255 (3.0%)	
Intersection	Yes	653 (7.7%)	2184 (25.8%)	1599 (18.9%)	4436 (52.5%)	.000***
	No	621 (7.3%)	1762 (20.8%)	1637 (19.4%)	4020 (47.5%)	
The time of crash	Morning	211 (2.5%)	485 (5.7%)	354 (4.2%)	1050 (12.4%)	.000***
	Afternoon	335 (4.0%)	1136 (13.4%)	1076 (12.7%)	2547 (30.1%)	
	Evening	262 (3.1%)	1139 (13.5%)	951 (11.2%)	2352 (27.8%)	
	Night	466 (5.5%)	1187 (14.0%)	856 (10.1%)	2509 (29.7%)	
Light condition	Sunny	659 (7.8%)	2586 (30.6%)	2270 (26.8%)	5515 (65.2%)	.000***
	Dusk	23 (0.3%)	61 (0.7%)	46 (0.5%)	130 (1.5%)	
	Down	63 (0.7%)	92 (1.1%)	84 (1.0%)	239 (2.8%)	
	Dark with good light	261 (3.1%)	536 (6.3%)	353 (4.2%)	1150 (13.6%)	
	Dark with poor light	56 (0.7%)	327 (3.9%)	234 (2.8%)	617 (7.3%)	
	Dark	212 (2.5%)	345 (4.1%)	250 (3.0%)	807 (9.5%)	
Air condition	Good air	1111 (13.1%)	3527 (41.7%)	2860 (33.8%)	7498 (88.6%)	.000***
	Foggy	41 (0.5%)	50 (0.6%)	81 (1.0%)	172 (2.0%)	
	Cloudy	55 (0.7%)	72 (0.9%)	66 (0.8%)	193 (2.3%)	
	Rainy	67 (0.8%)	298 (3.5%)	230 (2.7%)	595 (7.0%)	
The season of crash	Autumn	321 (3.8%)	1079 (12.8%)	846 (10.0%)	2246 (26.6%)	**.046**
	Winter	341 (4.0%)	898 (10.6%)	776 (9.2%)	2015 (23.8%)	
	Spring	295 (3.5%)	882 (10.4%)	765 (9.0%)	1942 (23.0%)	
	Summer	317 (3.7%)	1088 (12.9%)	850 (10.0%)	2255 (26.7%)	

Significance: Codes: 0 ‘***’ 0.001 ‘**’ 0.01 ‘*’ 0.05 '.'

### Injury severity levels

Most victims 3947 (46.7%) and 3237 (38.3%) had severe and fatal injuries, respectively (Table 3).

### Descriptive statistics

An association between the injury severity levels and associated factors is shown in Table 3 using the chi-square test of association. Driver’s sex and age, driving experience, day of crash, type of crash, type of vehicle (two or three wheelers, public transport, Commercial truck, automobile, Others), vehicle ownership (private or government), division of road (round about, one lane, two lanes and others), and air condition (good air, foggy, cloudy and rainy) were found to be significantly associated with the severity level of crash injuries (Table 3).

In the current study, a test of proportional odds assumption or a non-significance test was performed to check that this assumption is not violated. This is confirmed in Table 1, where we accepted the null hypothesis, which assumes that the parameters are the same for all categories. From Table 1, the chi-squared statistic is 15,024.84 with 8400 degrees of freedom. The *p*-value for this test was 0.217, which is not significant. This implies that there is no evidence to suggest that the parameters differ for each category. In other words, the assumption of proportional odds is satisfied (Table 1).

On the other hand, other diagnostics that are used to detect a lack of fit can be seen in Table 2. The first row displays the values of Pearson chi-square statistics computed by a covariate pattern. A small *p*-value is an indication that something is wrong with the model. Yet, the reported *p*-value 0.897 compared with a value of 0.05 showed that the overall model is fit. A 5% significance level was used since our sample size is larger (n > 400) [27, 33].

Same as deviance X^2^ in the second row of the same Table 2. This means that there is no evidence to suggest that the model does not fit the data at the 0.05 level of significance (Table 2).

**Fig. 1:**
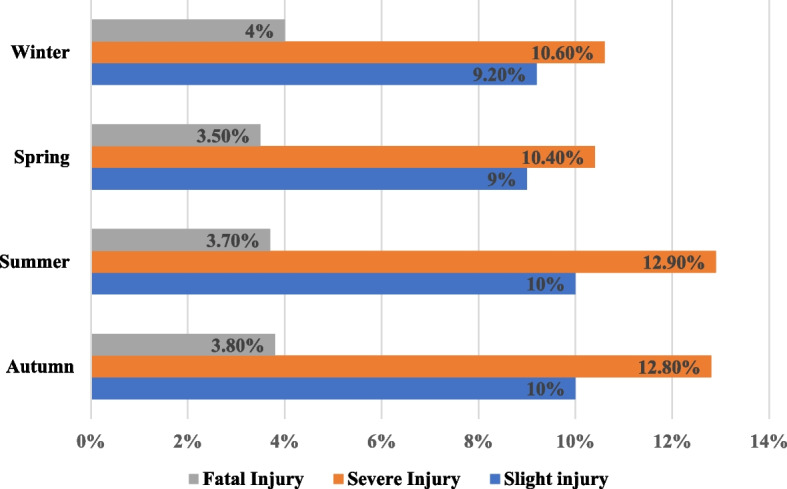
Road traffic injury severity levels in different seasons in Addis Ababa, Ethiopia, from October 2017-July 2020

From the results, most accidents 2246 (26.6%), 2015 (23.8%), 1942 (23%), and 2255 (26.7%) were occurring in autumn, winter, spring, and summer seasons, respectively (Fig. 1).

### Ordered logistic regression analysis

In this section, ordinal logistic regression analysis was done to test the association between explanatory variables, and outcome variables (injury severity levels of the road traffic accidents).

Accordingly, the day of crash, drivers’ education and driving experience, type of crash, type of vehicles, vehicle movement, owner of the vehicle, victim of crash, division of road and types of roads, time of crash, sub-city of the accident, and light condition were included in the model.

From the result of an estimated coefficient of ordered logistic regression analysis, log of odds was converted into an odds ratio for ease understanding (Tables 4 and 5).

**Table 4:** Factors affecting road traffic accident injury severity levels in Addis Ababa, Ethiopia, from October 2017- July 2020

Variables	**Category**	**Coefficient**	**Odds ratio**	**Stand. error**	**z-value**	***P*-value**
Driver sex	Male (Ref)					
	Female	-0.093	0.912	0.068	-1.379	0.168
Driver’s age	< 18 years (Ref)					
	18–30 years	0.175	1.373	0.060	2.904	0.004**
	31–50 years	0.461	1.349	0.064	7.244	0.000***
	> 50 years	0.412	1.282	0.068	6.098	0.000***
Driver educational level	High school and lower (Ref)					
	College and above	0.369	1.447	0.048	7.664	0.000***
Driving experience	1–2 years	-0.203	0.816	0.060	-3.401	0.001***
	3–5 years	-0.044	0.957	0.069	-0.637	0.524
	6–10 years	-0.373	0.689	0.078	-4.801	0.000***
	> 10 years	-0.477	0.620	0.070	-6.789	0.000***
Day of crash	Monday (Ref)					
	Tuesday	0.065	1.067	0.080	0.807	0.420
	Wednesday	0.028	1.029	0.080	0.353	0.724
	Thursday	0.072	1.075	0.078	0.924	0.355
	Friday	0.455	1.576	0.088	5.14	0.000***
	Saturday	-0.004	0.996	0.078	-0.048	0.961
	Sunday	0.182	1.200	0.079	2.307	0.021*
The type of crash	The crash with pedestrian (Ref)					
	Crash with another vehicle	-0.287	0.751	0.094	-3.044	0.002**
	Roll over	0.386	1.471	0.143	2.698	0.007**
	Crash with animal or objects	-0.033	0.967	0.132	-0.25	0.803
	Others	0.342	1.408	0.175	1.955	0.051
The type of vehicle	Two or three wheelers (Ref)					
	Public transport	0.247	1.280	0.074	3.359	0.001***
	Commercial truck	0.464	1.590	0.077	6.036	0.000***
	Automobile	0.111	1.117	0.079	1.398	0.162
	Others	1.355	3.878	0.148	9.179	0.000***
The owner of the vehicle	Private (Ref)					
	Government	0.192	1.211	0.070	2.725	0.006**
	Others	0.143	1.154	0.097	1.483	0.138
Movement of vehicle	Straight (Ref)					
	Curved	0.216	1.241	0.068	3.181	0.001**
	Reverse	-0.072	0.930	0.127	-0.569	0.569
	Parked	0.313	1.368	0.189	1.662	0.096
	Others	1.067	2.906	0.195	5.474	0.000***
Victims of the crash	Driver (ref)					
	Passenger	-0.360	0.698	0.092	-3.889	0.000***
	Pedestrian	-0.347	0.418	0.049	-3.752	0.000***
	Motorcyclist	-0.456	0.634	0.117	-3.895	0.000***
	Motorcycle passenger	0.369	1.447	0.145	2.541	0.011*
Sex of the victims	Male (Ref)					
	Female	-0.064	0.938	0.049	-1.315	0.189
Movement of pedestrian	Crossing (Ref)					
	Walking	-0.281	0.755	0.048	-5.849	0.000***
	Sitting	-0.110	0.896	0.092	-1.2	0.230
	Other	3.285	26.708	0.215	15.248	0.000***
Division of roads	Round about					
	One lane	-0.542	0.582	0.086	-6.33	0.000***
	Two lanes	-0.432	0.649	0.058	-7.502	0.000***
	Others	-0.452	0.636	0.086	-5.248	0.000 ***
Sub-city of accidents	Akaki Kality (Ref.)					
	Arada	0.326	1.385	0.121	2.686	0.007**
	Nifas Silk	-0.467616	0.626	0.088	-5.325	0.000***
	Bole	0.845	2.328	0.108	7.809	0.000***
	Yeka	0.569	1.767	0.119	4.801	0.000***
	Kolfe Keraniyo	0.162	1.176	0.099	1.635	0.102
	Addis Ketema	-0.497	0.608	0.114	-4.355	0.000***
	Kirkos	-0.228	0.796	0.106	-2.156	0.031*
	Gulele	-1.079	0.340	0.130	-8.277	0.000***
	Lideta	-0.008	0.992	0.114	-0.066	0.947
Type of road	Straight (Ref)					
	Zigzag	0.607	1.835	0.238	2.549	0.011*
	Curve	-1.027	0.358	0.079	-12.979	0.000***
	Tilted	0.118	1.125	0.123	0.959	0.338
	Hill	0.021	1.021	0.135	0.152	0.879
Intersection	Yes	1				
	No	0.045	1.046	0.047	0.949	0.343
Time of crash	Morning	1				
	Afternoon	-0.192	0.825	0.076	-2.53	0.011*
	Evening	-0.201	0.818	0.077	-2.618	0.009**
	Night	-0.238	0.788	0.079	-3.009	0.003**
Light condition	Sun light (ref)					
	Dusk	0.143	1.154	0.180	0.798	0.425
	Down	0.497	1.644	0.143	3.482	0.000***
	Dark with good light	0.315	1.370	0.074	4.257	0.000***
	Dark with poor light	0.141	1.151	0.089	1.589	0.112
	Dark	0.532	1.703	0.088	6.069	0.000***
Air condition	Good air (Ref)					
	Foggy	-0.126	0.881	0.163	-0.773	0.439
	Cloudy	0.242	1.274	0.152	1.597	0.110
	Rainy	-0.148	0.862	0.091	-1.63	0.103
Model summary	The number of obs. = 8458					
	Log likelihood = -7458					
	LR Stat = 2141.8					
	Prob > Chisq. = < 0.000					
	Pseudo.R2 = 0.258053					

Significance Codes: 0 ‘***’ 0.001 ‘**’ 0.01 ‘*’ 0.05 ‘.’ 0.1 ‘ ’ 1

**Table 5:** Odd ratio coefficients and confidence intervals

**Variables**	**Odds ratio (OR)**	**2.50%**	**97.50%**
Day of crash (Tuesday)	1.0670185	0.9114879	1.2490895
Day of crash (Wednesday)	1.0285098	0.8799933	1.2020852
Day of crash (Thursday)	1.0748643	0.9222735	1.2527567
Day of crash (Friday)	1.5757825	1.3250291	1.8743219
Day of crash (Saturday)	0.9962256	0.8546085	1.1613134
Day of crash (Sunday)	1.1998547	1.0278045	1.4008206
Sex of driver (Female)	0.9107994	0.7974243	1.0400533
Drivers age (18–30)	1.3727057	0.9442592	2.0089782
Drivers age (31–50)	1.3493279	0.9243851	1.982567
Drivers age (> 50)	1.2816181	0.8520256	1.9385618
Drivers education (College or Higher)	1.4466626	1.3163433	1.5900098
Driving experience (1–2 years)	0.8162527	0.7261027	0.9175337
Driving experience (3–5 years)	0.9571962	0.8365731	1.0951467
Driving experience (6–10 years)	0.6887653	0.5914094	0.8018935
Driving experience (> 10 years)	0.6204448	0.5404911	0.7120265
Type of Crash (with another vehicle)	0.7505783	0.6238828	0.902793
Type of Crash (rollover)	1.4707633	1.1112244	1.9465834
Type of Crash (with animal or another objects	0.9674765	0.7464024	1.2534711
Type of Crash (others)	1.4084002	0.9986771	1.9851529
Type of vehicle (public transport)	1.2801839	1.1084987	1.4788312
Type of vehicle (commercial truck)	1.5900125	1.3679529	1.8486979
Type of vehicle (automobile)	1.1171736	0.9565244	1.3050026
Type of vehicle (others)	3.8779902	2.9062183	5.1855427
Vehicle movement (Curved)	1.2409092	1.0863307	1.4173914
Vehicle movement (Reversed)	0.9303322	0.7250266	1.1922335
Vehicle movement (Parked)	1.3679919	0.9451434	1.9800594
Vehicle movement (Others)	2.9062131	1.9821735	4.2577936
Owner of vehicle (Government)	1.2112346	1.0552766	1.3902479
Owner of vehicle (Others)	1.1539536	0.9548353	1.3941274
Sex of the victim (Female)	0.9377684	0.8520753	1.0320179
Victim of Crash (Passenger)	0.6979028	0.5821186	0.8365104
Victim of Crash (Pedestrian)	0.4182341	0.0741420	0.6441236
Victim of Crash (Motorcyclist)	0.6337555	0.5035697	0.7970003
Victim of Crash (Motor Passenger)	1.4469693	1.0879458	1.924064
Movement of Pedestrian (Walking)	0.7550037	0.6871077	0.8295289
Movement of Pedestrian (Sitting)	0.8958351	0.7483406	1.0719693
Movement of Pedestrian (Others)	26.7077351	17.7761326	41.473491
Division of Roads (One-lane)	0.5817347	0.4917508	0.6877933
Division of Roads (Two-lane)	0.649385	0.5800752	0.7268668
Division of Roads (Others)	0.6361937	0.5371557	0.7530616
Sub-city (Arada)	1.3854787	1.0921485	1.7578805
Sub-city (Nifas Silk)	0.6264939	0.5274046	0.7441531
Sub-city (Bole)	2.327594	1.883444	2.8783349
Sub-city (Yeka)	1.7667364	1.4006657	2.2293181
Sub-city (Addis Ketema)	0.6081875	0.4861241	0.7606291
Sub-city (Kirkos)	0.7962629	0.6472333	0.9794897
Sub-city (Gulele)	0.3398853	0.2628706	0.4382962
Types of Roads (Zigzag)	1.8349143	1.1505014	2.9313126
Types of Roads (Curve)	0.358147	0.3065139	0.4179815
Types of Roads (Tilted)	1.1249262	0.884302	1.4311951
Types of Roads (Hill)	1.0208318	0.7828518	1.3309666
Intersection (No)	1.045953	0.9532757	1.1476846
Time of Crash (Afternoon)	0.8250475	0.7108214	0.9576192
Time of Crash (Evening)	0.8182474	0.7041307	0.9508512
Time of Crash (Night)	0.7882362	0.6750236	0.9203746
Light condition (Dusk)	1.1540668	0.8109149	1.6405374
Light condition (Dawn)	1.6443737	1.2428962	2.1760718
Light condition (Dark-with-good-light)	1.3700735	1.1851966	1.5838629
Light condition (Dark-with-poor-light)	1.1511666	0.9675196	1.3693061
Light condition (Dark)	1.7030493	1.4341499	2.0228378
Air condition (Foggy)	0.8812625	0.6386112	1.2127082
Air condition (Cloudy)	1.2739825	0.9463198	1.7151791
Air condition (Rainy)	0.8620238	0.7209051	1.0302888

The odds of a crash injury being fatal/severe /slight is 1.57 times higher for Friday holding Monday as reference. The confidence interval for odds could be as minimum as 1.325 and as maximum as 1.874 with 95% confidence and shows that it is statistically significant since it excludes one.

The results shows that the odd of the crash being fatal/severe/slight injury is 1.446 times higher among college or higher-level educated drivers than among less educated drivers. The 95% confidence interval also suggests that the odd could be as minimum as 1.316 and as maximum as 1. 590. This could seem as a contradictory finding, which could happen for some reasons.

These possible reasons include compromising some of the driving ethics and traffic rules such as over speeding to arrive on time for meetings and other duties, working by phone while driving, and being engaged in busy work by well-educated drivers, which could make them spend a long time on the phone while driving.

The odds of a crash injury being fatal/severe is 2.32 times higher in Bole and 1.76 times higher in Yeka sub-cities than accidents happened in Akaki kality sub-city for some reasons. Both of these sub-cities have large residential and commercial areas with the number of cross-roads and large square areas congested with passengers and vehicles that could make these areas spot for road traffic accidents.

According to the result of the analysis, individuals who sustained rollover type of crash injuries were about 1.470 times more likely to have a fatal injury than their counterparts, OR 1.470 (95% CI; 1.111- 1.946). And also, the odds of the crash injury being fatal or severe among crash victims by dump truck, ambulance, vans, crane truck, and others were nearly four times more likely than two or three-wheeler vehicles, OR 3.877 (95% CI: 2.906–5.185) (Tables 4 and 5).

The odds of fatal or severe injury for individuals who had sustained a crash injury on the zigzag type of the roads were found to be nearly two times more likely as compared to the injury sustained on the straight road, OR 1.834 (95% CI: 1.150–2.931).

From the study, the division of roads was found to have a statistically significant association with crash injury severity levels. Division of roads into one-lane, two-lane, and others is about 0.581, 0.649, 0.636 times less likely to result in fatal/severe injuries as compared to a roundabout road. Being the passenger of the vehicle has a lower odd of 0.697 times resulting in fatal/severe injury as compared to the driver of the vehicle (Tables 4 and 5).

Table 5 shows the odds ratio coefficients and confidence intervals of the variables associated with road traffic accidents injury severity levels in Addis Ababa, Ethiopia (Table 5).

## Discussion

This study was conducted to identify the major risk factors associated with the injury severity levels induced by road traffic accidents in Addis Ababa. All recorded road traffic accidents from October 2017 to July 2020 were included in the study. The ordinal logistic regression model was found to be the most suitable model and was used in this current study for conducting ordinal data analysis [24, 29].

It was indicated in the current study that the age of the driver was found to be statistically significant in determining the road traffic accident’s injury severity levels. Young drivers increase injury severity in road traffic accidents. This could be due to the desire to drive at high speed by young drivers, overconfidence, liability to distracted driving, and from less experience. This impact of driver age on the injury severity level has also been confirmed in previous studies [34, 35].

The findings of this study showed that 6039 (71.4%) of the total road traffic accidents happened to pedestrians. Out of these accidents, 1017 (12.0%) were fatal and 2862 (33.8%) were severe injuries. This could be attributed to the fact that pedestrians are less protected than passengers of the vehicle, poor adaptation to the road traffic rules, and lack of adequate crossings for pedestrians. The result of the study done in Tanzania showed that passengers are the dominant victims of road traffic accidents, contrary to the finding of the current study [36]. This discrepancy might be due to the violent behavior of the drivers, poor enforcement of the traffic rules on the road safety practice and less awareness among pedestrians on how they should behave on the roads.

Regarding the age of the road traffic accident victims, majority 2680 (31.7%) of them were within the age group of 18–30 years. This could be due to the fact that younger road users were the predominant group of the community in the study area, lack of pedestrian routes, and convenient public transportation. This finding was in line with previous studies done in Ethiopia so far [6, 37].

This study identified that the day of the week on which severe road traffic accident injuries occurred the most was Friday, followed by Sunday. This is in contrary to the study finding in Ghana, where Saturday was the day on which severe road traffic accident injuries most commonly occurred [16, 38]. This difference might be attributed to cultural and societal values, and activities being held on these days may vary from country to country. For instance; in Ethiopia, since Friday is the end of the week, private and government employees might travel from different work places to Addis Ababa to spend their weekend with their family, which can result in traffic congestion and can increase injury severity level. Furthermore, Sunday is the most popular day for various gatherings, such as weddings, religious programs, football games, and celebrations, which could increase the risk of drunk driving, and sustaining severe crash injuries.

Regarding the educational status of the driver, the probability of sustaining fatal or severe injury was 1.446 times higher among college-or higher level-educated drivers than among less educated drivers in the current study, OR 1.446 (95% CI; 1.316–1.590). However, this is different from the study conducted by Lidetu A, et al.in the Gamo Gofa zone, Southern Ethiopia, which stated that the majority of the fatal and severe crash injuries were from collisions by less educated drivers [12]. Possible reasons for this difference might be attributed to study setting and study period differences. On another point of view, compromising some of the driving ethics and traffic rules such as over speeding by well-educated drivers to arrive on time for meetings and other duties, might be common in the Addis Ababa city, which is the metropolitan city of Ethiopia and the seat of the African Union.

Moreover, this study revealed that commercial truck was found to have a significant impact, OR 1.590 (95% CI;1.368–1.849) on determining the RTI severity level, which was in agreement with previous studies [8, 39]. The possible explanation for this could be that the weight of the trucks is much greater, and even minor accidents can cause severe to fatal injuries. Trucks are larger and heavier than other vehicles, and drivers may be unable to stop their trucks quickly if they are driving very fast, which can cause severe to fatal injuries.

On the other hand, rollover (vehicle upside down) type of crash was likely to increase the probability of severe or fatal injuries among the victims of the road traffic accidents than vehicle -pedestrian collisions. This is similar to the findings of the previous studies reported in the literatures [40]. The possible reasons for this could be, those occupants who were not using restraints were more likely to be ejected from a rolling vehicle, and those who were ejected were more probably sustain severe or fatal injuries. In addition, drivers who did not attempt an avoidance maneuvre (braking or steering), and driving over allowed speed have the propensity to rollover crash that could cause severe or fatal crash injuries. This is because when a vehicle rolls over, it can crush the occupants inside by vehicle’s bodies and structures, since vehicles usually landed up on their side or roof [41].

The present study also considered that a government-owned vehicle has a significant impact (OR = 1.211) on the distribution of the probability of the severe or fatal crash injuries to the collision victims, which agreed with a study done in Ethiopia [22]. This could possible because of poor handling and utilization of the government-owned vehicles by the drivers in the study area.

The proportion of collisions occurring during curved maneuvers of the vehicle either to the left or right is likely to increase the probability of severe or fatal injuries as compared to the straight movement of the vehicle OR = 1.240 (95% CI:1.086,1.417). This was contrary to the study done in Singapore [20], which showed that an accident occurred while vehicle movement was either to the left or right had a lower probability of severe or fatal injuries. This difference might be attributed to the fact that the study participants being studied in Singapore were only involved in an out-of-control single vehicle crash.

This study’s findings showed that the probability of severe or fatal injuries from road crash accidents occurring during the evening and nighttime was significantly lower than the probability of severe or fatal injuries occurring in the morning, OR = 0.818 (95% CI: 0.704, 0.950) and OR = 0.788 (95% CI: 0.675, 0.920), respectively. This agreed with the findings of the study conducted in the Oromia region of Ethiopia [40]. In contrast, the current study identified that the probability of severe or fatal injuries was lower in crash accidents occurring during the afternoon, which opposed the findings of the study done in the Oromia region [40], but was in agreement with the study done in China [8].

Furthermore, some of the previous studies reported that the time of the crash was not statistically significant in the determination of the crash injury severity levels [21].However, there was another study that reported that the risk of death was higher among victims who sustained injuries between midnight and dawn (6 a.m.) than at other times of the day [42]. These findings are in disagreement with current study results. These discrepancies could be attributed to the differences of the study settings, like having a varying number of working shifts among communities of study areas, the culture of spending longer night hours at parties and drunk driving home after party attendance, and the availability of roadside lighting.

The present study also assessed the sub-cities that are prone to road traffic accidents and those that are more susceptible to severe to fatal crash accidents within the study area. Out of the ten sub-cities, Bole, Yeka and Arada sub-cities have a higher probability of severe or fatal injuries than Akaki Kality and other sub-cities, with OR = 2.32 (95% CI: 1.88, 2.87), OR = 1.76 (95% CI: 1.40, 2.22), and OR = 1.38 (95% CI: 1.092, 1.75), respectively. In these areas, heavy traffic volumes, crowds of passengers, intersections, numerous traffic signals, and mixed land use patterns for commercial and residential purposes probably increased crash accident occurrences and injury severity levels. This is in agreement with the study done in Hong Kong [43]. However, in contrast to the findings of another study conducted in Hong Kong, which indicated that commercial areas are safer than residential areas. This was because there were more pedestrian overpasses that separated motor vehicles from pedestrians who were crossing the street, and there were a lot of ground-floor shops in Hong Kong’s residential areas. Unfortunately, such road structure and building alignments with respect to the pedestrian’s road are scarce in Addis Ababa’s commercial areas, and the number of crash accidents in these areas is higher than in other areas.

The crash accidents occurring under dark light conditions in the environment had a significant influence on the severity of road traffic injuries compared with crash accidents occurring under good light conditions in Addis Ababa, with OR = 1.703 (95% CI: 1.434, 2.022). This was in agreement with previous studies [20, 35]. A possible explanation for this is that reduced traffic volumes after dark could lead to increased vehicle speeds, which are likely to increase the risk of a collision and the severity of injury to the victim of the crash. Additionally, drivers are more likely to be inebriated after dark and may also feel sleepier and drowsier, which increases the likelihood that they will be involved in a road traffic accident and sustain severe crash injuries [44–46].

### Limitations of the study

This study depends on secondary information. Thus, injury severity-level categorizations were considered as they were registered on the road traffic accident records of the Addis Ababa police commission. Also, some very important data, such as seat belt and helmet utilization, drunk driving, the number of people who died from crashes per accident, and geometric and traffic information, were missing. Likewise, the vehicle’s GPS data, which could help to know the correct spot of an accident, the speed of the vehicle, and the number of passengers in the vehicle per accident, were not available. As a result, we were unable to estimate traveled vehicle hours and specific time-related estimations.

## Conclusion

From the total crash victims, pedestrians share the highest number of severe to fatal crash injuries, mainly in the summer and autumn seasons. Moreover, younger drivers who were found in the age group of 18–30 years old were found to be more commonly affected than other victims of crash injuries. This study found that severe to fatal crash injuries were attributed to commercial trucks, government-owned vehicles, college and above level educated drivers, rollover crashes, curved maneuver of vehicles, motorbike passenger, crash day on Friday, and darkness. It is recommended that the city traffic police office should give special consideration to commercial truck and public transport drivers, motorcyclists, and all other drivers who drive without adequate lighting. However, to make these police control measures much more effective, it is very essential to complement them with specific communication campaigns and in-service training programs for traffic police, increasing community awareness of road safety and appropriate behaviors on the road through various media campaigns [47, 48].

Furthermore, attention has to be given to the handling of the government vehicle by the respective government offices to which these vehicles are affiliated, and licensing authorities must implement robust assessment procedures before qualifying drivers. In addition, this study found that the occurrence of traffic accidents and the probability of severe to fatal crash accidents are commonly occurring in the morning. As a result, much more attention and extensive road safety control measures have to be implemented to reduce the impacts of road crash accidents. Also, mixed-use land (i.e., commercial and residential) areas in the city have been sustaining a larger number of crash accidents and severe to fatal injuries. Hence, the road traffic department of the city has to consider the establishment of additional pedestrian overpasses in highly overcrowded areas, which would separate street-crossing pedestrians from vehicles on the road to reduce the occurrence of road traffic accidents. Lastly, the current study established that crash accidents occurring in dark light conditions highly contributed to crash injury severity levels compared with crash accidents occurring in good light conditions. It would be beneficial if roadway lighting or illumination were established in crash-prone areas of the city.

## Data Availability

The datasets used during this study are available from the corresponding author on reasonable request.

## Abbreviations

ABO: Ararso Baru Olani
AW: Asfawosen Woldemeskel
CI: Confidence Intervals
GDP: Gross Domestic Product
GG: Geleta Guta
M: Mean
MA: Micheal Alemayehu
OR: Odds Ratio
Ref: Reference
RTI: Road Traffic Injuries
SD: Standard Deviation
TB: Tariku Bekelcho
USD: United States Dollar
